# Sensitivity for Malignancy with Navigation Bronchoscopy in Small Lung Nodules

**DOI:** 10.3390/arm94050067

**Published:** 2026-09-17

**Authors:** Anders Näslund, Melker Ottosson, Marija Karadzovska-Kotevska, Karl Teurneau-Hermansson, Jaroslaw Kosieradzki, Stefan Barath, Hamid Akbarshahi

**Affiliations:** 1 Department of Clinical Sciences Lund, Division of Respiratory Medicine, Allergology, and Palliative Medicine, Lund University, SE-221 85 Lund, Sweden; melker.ottosson@skane.se (M.O.); hamid.akbarshahi@med.lu.se (H.A.); 2 Department of Respiratory Medicine and Allergology, Skåne University Hospital, SE-221 85 Lund, Sweden; marija.kotevska@med.lu.se (M.K.-K.); jaroslaw.kosieradzki@skane.se (J.K.); stefan.barath@skane.se (S.B.); 3 Department of Clinical Sciences Lund, Division of Oncology, Lund University, SE-221 85 Lund, Sweden; 4 Department of Cardiothoracic Surgery, Skåne University Hospital, SE-221 85 Lund, Sweden; karl.teurneau-hermansson@med.lu.se; 5 Department of Clinical Sciences Lund, Division of Cardiothoracic Surgery, Lund University, SE-221 85 Lund, Sweden

**Keywords:** electromagnetic navigation bronchoscopy, pulmonary nodules, sensitivity, bronchus sign, lung cancer diagnosis

## Abstract

**Highlights:**

**What are the main findings?**

- Sensitivity for malignancy with electromagnetic navigation bronchoscopy (ENB) was markedly lower in nodules ≤10 mm than in larger lesions.
- Lesion size and bronchus sign were independently associated with diagnostic sensitivity.

**What are the implications of the main findings?**

- ENB should be considered selectively in very small nodules and interpreted alongside pretest probability and anatomy.
- Surveillance, surgery, or newer bronchoscopic platforms may be appropriate depending on risk, accessibility, and local resources.

**Abstract:**

Electromagnetic navigation bronchoscopy (ENB) is used for minimally invasive assessment of peripheral pulmonary nodules, but data on lesions ≤10 mm are limited. When ENB is performed for suspected lung cancer, the key clinical question is whether malignancy is detected rather than whether any diagnosis is obtained. For this reason, sensitivity for malignancy was selected over diagnostic yield as the primary outcome. We retrospectively analyzed 541 consecutive ENB procedures performed at Skåne University Hospital during 2018–2019 and followed patients for at least two years to determine final diagnosis. Overall, 323 lesions (59.7%) were malignant and median lesion diameter was 13 mm. Sensitivity for malignancy was 44.0% overall but was markedly lower in lesions ≤10 mm than in larger lesions (23.3% vs. 50.0%, *p* < 0.001). Larger lesion size and the presence of a bronchus sign were independently associated with higher sensitivity, whereas lower lobe location was associated with reduced sensitivity. In very small nodules, the low probability of obtaining a malignant diagnosis with ENB should be considered when selecting patients and weighing ENB against surveillance, surgery, or alternative diagnostic approaches.

## 1. Introduction

Invasive diagnostic evaluation of small and peripheral pulmonary nodules remains a clinical challenge. This challenge has become increasingly relevant in the context of rising nodule detection rates driven by widespread use of chest computed tomography (CT) and lung cancer screening programs [1,2]. Available diagnostic approaches include surgical biopsy, CT-guided transthoracic needle biopsy (TTNB), and advanced bronchoscopic techniques such as electromagnetic navigation bronchoscopy (ENB). ENB is an established bronchoscopic technique that has been increasingly adopted for the evaluation of peripheral pulmonary nodules. More recently, robotic-assisted bronchoscopy has emerged as a diagnostic option with impressive results. However, its availability remains limited.

ENB is a minimally invasive bronchoscopic technique that uses electromagnetic guidance and a virtual three-dimensional bronchial map generated from a preprocedural chest CT scan to navigate a catheter through peripheral airways toward a target lesion [3]. The procedure allows sampling of peripheral lung nodules and enables biopsy of multiple lesions as well as mediastinal lymph node staging during the same session, making it a versatile tool in the diagnostic work-up of suspected lung cancer.

According to published studies, ENB has demonstrated a favorable safety profile compared with TTNB, with lower complication rates in pooled analyses [4,5,6]. Diagnostic yield in large cohorts and meta-analyses typically ranges between 65 and 75% [6,7]. As with other bronchoscopic techniques targeting peripheral lesions, outcomes are strongly influenced by lesion-related factors, particularly lesion size and the presence of a bronchus sign on CT imaging [7]. Despite this overall favorable performance, important uncertainties remain when ENB is applied to the smallest peripheral lung nodules, where data are very limited [8].

Most published ENB studies report diagnostic yield rather than sensitivity for malignancy. However, definitions of diagnostic yield vary substantially across studies and differ in their handling of false-negative results [9]. Sensitivity for malignancy more directly reflects the ability of the procedure to detect cancer and may therefore be particularly informative when ENB is performed for suspected malignancy. This distinction is especially relevant in very small pulmonary nodules, where the pre-test probability of cancer is lower [10]. To date, sensitivity for malignancy has not been specifically reported for lesions ≤10 mm, despite this subgroup representing substantial diagnostic uncertainty in clinical practice.

Against this background, we retrospectively analyzed 541 ENB procedures performed at Skåne University Hospital, Lund, during 2018 and 2019, including 162 procedures targeting lesions ≤10 mm. The primary aim of this study was to evaluate sensitivity for malignancy in very small peripheral lung nodules, with secondary analyses according to bronchus sign status, in order to refine patient selection and procedural decision-making for ENB in this challenging subgroup. We hypothesized that sensitivity for malignancy would be significantly lower in lesions ≤10 mm compared to lesions >10 mm.

## 2. Materials and Methods

### 2.1. Study Design and Data Sources

This was a retrospective, single-center observational study including all patients who underwent ENB at Skåne University Hospital, Lund, Sweden, during the years 2018 and 2019. The cohort was originally compiled for internal clinical quality control and was later expanded with additional data when the decision was made to use it for research. Data were retrieved from electronic medical records, radiology information systems, regional electronic patient databases, and the Region Skåne pathology database. All patients were followed for a minimum of two years after the ENB procedure to ascertain final diagnostic outcome. This study was conducted and reported in accordance with the Strengthening the Reporting of Observational Studies in Epidemiology (STROBE) guidelines [11].

### 2.2. Study Population

All consecutive ENB procedures during the study period were included, reflecting clinical practice. Patients in whom the ENB procedure was initiated but not completed were excluded. No additional exclusion criteria were applied.

### 2.3. Procedure

Procedures were performed using the SuperDimension™ electromagnetic navigation system (Medtronic, Minneapolis, MN, USA), with fluoroscopy and radial endobronchial ultrasound (Olympus Medical Systems Corp., Tokyo, Japan) used in all cases. Tissue sampling was performed using biopsy forceps, cytology brush, and suction catheter. Cryobiopsy was not used. Rapid on-site evaluation (ROSE) was used when available. All procedures were conducted by one of three experienced operators with established expertise in ENB and were performed under conscious sedation with midazolam and alfentanil, with spontaneous breathing maintained throughout.

### 2.4. Definitions and Measurements

Lesion size was defined as the mean diameter, calculated from the maximum diameter measured on thin-section axial CT images (slice thickness ≤ 1 mm) and its largest perpendicular diameter obtained using multiplanar reconstructions. The largest lesion was used when multiple targets were sampled. A positive bronchus sign was defined as bronchus sign types Ia, Ib, Ic, or IIa according to the classification proposed by Imabayashi et al. [12]. Bronchus sign status was assessed by the lead author, who was blinded to clinical outcomes. FDG-PET findings were classified based on the clinical nuclear medicine report. Lesions described as metabolic but not hypermetabolic were considered PET-negative, and those with at least mild hypermetabolism were considered PET-positive, without a fixed SUV threshold.

### 2.5. Outcomes

The primary outcome was sensitivity for malignancy. True positive cases were defined as lesions with a malignant diagnosis established by ENB sampling. True negative cases were defined as lesions with a negative ENB result and any of the following during follow-up: at least two years of radiological follow-up without lesion progression; significant radiological regression of the lesion; or surgical resection demonstrating benign pathology.

False negative cases were defined as lesions with a negative ENB result followed within two years by surgical resection showing malignancy; radiotherapy directed at the target lesion without histological confirmation; an alternative diagnostic procedure confirming malignancy of the target lesion; a subsequent lung cancer diagnosis considered clinically or radiologically to correspond to the target lesion; or an extrathoracic malignancy where the target lesion was considered consistent with pulmonary metastatic disease. Classification was based on case-by-case review of the clinical and radiological record.

False positive cases were not considered. Indeterminate cases included patients who died from unrelated causes during follow-up or were lost to follow-up due to relocation outside the region or referral from external healthcare providers without accessible follow-up data.

Secondary outcomes included the effect of bronchus sign status on sensitivity and frequency of adverse events. Additional variables collected included inclusion in the standardized lung cancer diagnostic pathway, use of ROSE, presence of hypermetabolism on PET imaging, lesion attenuation (solid vs. subsolid), and lobar location.

### 2.6. Statistical Analysis

Sensitivity for malignancy was calculated as the proportion of true positive cases among all malignant cases with a definitive outcome (true positives plus false negatives). Indeterminate cases were excluded from sensitivity analyses.

A comparison of diagnostic sensitivity was performed between lesions ≤10 mm and lesions >10 mm, with exact binomial 95% confidence intervals calculated. Sensitivity was also compared across predefined lesion size categories (≤10 mm, 11–20 mm, and >20 mm). Differences in sensitivity between groups were assessed using the chi-square test.

To explore independent predictors of diagnostic sensitivity, multivariable logistic regression analysis was performed among malignant lesions. Lesion size was modeled as a continuous variable, and bronchus sign, lobar location (lower vs. upper/middle), lesion density, FDG-PET avidity, use of ROSE, and sampling of more than one target lesion were included as covariates. Analyses were performed using complete case analysis. Results are presented as odds ratios with 95% confidence intervals. A two-sided *p*-value < 0.05 was considered statistically significant. Statistical analyses were performed using RStudio (R version 4.5.2; R Foundation for Statistical Computing, Vienna, Austria).

## 3. Results

A total of 541 ENB procedures were performed during 2018–2019. Baseline patient, lesion, and procedural characteristics are presented in Table 1. The median lesion diameter was 13 mm (IQR 10–17). The presence of a bronchus sign was highly size-dependent: it was present in only 11.1% (*n* = 18) of nodules ≤10 mm, compared to 30.7% (*n* = 92) in 11–20 mm nodules and 63.3% (*n* = 50) in lesions >20 mm.

After a minimum follow-up of two years, 323 lesions (59.7%) were classified as malignant, 162 (29.9%) as benign, and 56 (10.4%) as indeterminate. Indeterminate cases did not differ across size categories (*p* = 0.61).

The overall sensitivity for malignancy in the cohort was 44.0% (95% CI 38.5–49.6). When stratified according to the predefined threshold, sensitivity was significantly lower in lesions ≤10 mm compared with lesions >10 mm (23.3% [95% CI 14.2–34.6] vs. 50.0% [95% CI 43.6–56.4], *p* < 0.001). In a worst-case analysis treating all indeterminate cases as false negatives, sensitivity was lower across all size categories but remained lowest for lesions ≤10 mm (Supplementary Table S1). The basis for classification of true-negative and false-negative outcomes is shown in Supplementary Table S2.

**Figure 1:**
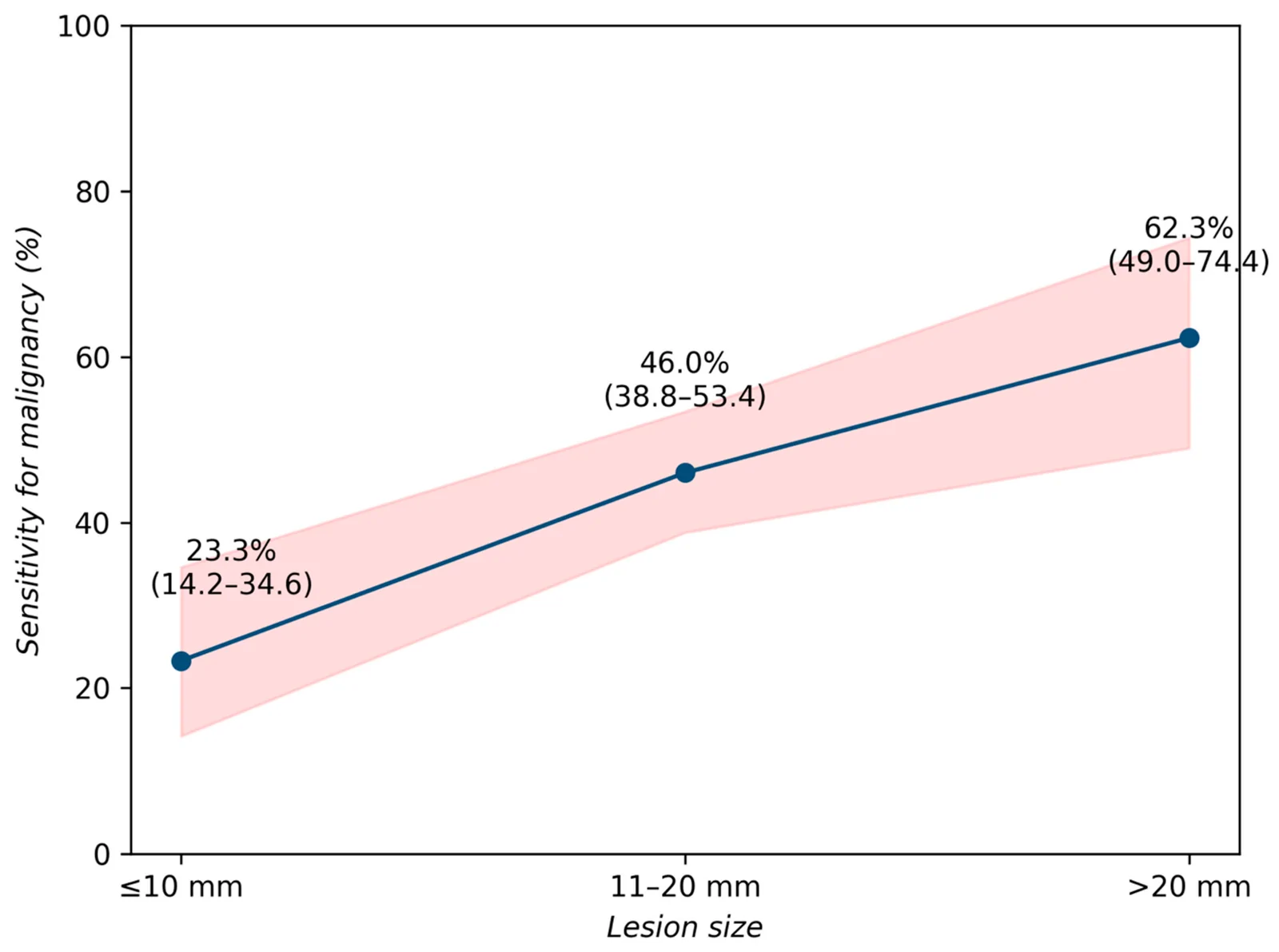
Sensitivity for malignancy according to lesion size category. Points indicate point estimates and the shaded area indicates 95% confidence intervals.

As seen in Figure 1, sensitivity increased progressively and significantly across predefined size categories (*p*-value for difference between adjacent groups: *p* = 0.001 for ≤10 mm vs. 11–20 mm, *p* = 0.039 for 11–20 mm vs. >20 mm).

Stratified analyses of lesion and procedural characteristics are shown in Table 2a. Sensitivity was significantly higher in lesions with a bronchus sign compared to those without (64.3% vs. 32.7%, *p* < 0.001) and in upper or middle lobe lesions compared with lower lobe lesions (48.1% vs. 35.8%, *p* = 0.0496), whereas procedural and radiographic variables, including the use of ROSE, sampling of >1 target lesion, and FDG-PET positivity, were not significantly associated with diagnostic sensitivity.

In the multivariable logistic regression analysis (Table 2b), lesion size was a significant independent predictor of higher diagnostic sensitivity, and the presence of a bronchus sign also emerged as an independent predictor of successful diagnosis. Conversely, lower lobe location was independently associated with reduced sensitivity. Other variables were not independently predictive. Multivariable analyses included 287 malignant lesions with complete data out of 323 malignant lesions.

Five procedure-related adverse events were recorded. Pneumothorax occurred in four cases (0.7%), three of which were treated with an ambulatory pleural drainage device and one managed with imaging follow-up only. One case of mediastinitis and empyema occurred following ENB performed concurrently with endobronchial ultrasound-guided transbronchial needle aspiration.

## 4. Discussion

### 4.1. Principal Findings

In this retrospective single-center study of ENB, we observed a significant reduction in sensitivity for malignancy in very small peripheral lung nodules (≤10 mm) compared with larger lesions. The primary study hypothesis was thereby confirmed. Lesion size was the main factor associated with diagnostic sensitivity. In addition, the presence of a bronchus sign was independently associated with higher sensitivity (aOR 2.53, *p* = 0.002), whereas lower lobe location was independently associated with reduced odds of a successful diagnosis (aOR 0.35, *p* < 0.001). These findings highlight important limitations of ENB performance in clinical practice when the technique is applied to the smallest peripheral pulmonary nodules, where diagnostic uncertainty is greatest.

### 4.2. Diagnostic Sensitivity in Context

The overall sensitivity for malignancy observed in this study (44.0%) was lower than that reported in many previous ENB publications. This difference is most plausibly explained by the small lesion size in the present cohort, with a median diameter of 13 mm and 29.9% of targeted lesions measuring ≤10 mm. Many prior studies, including large prospective cohorts such as NAVIGATE, included lesions with substantially larger median diameters, a factor consistently associated with higher diagnostic yield and sensitivity [7,13]. In addition, several studies have not reported sensitivity for malignancy, instead applying varying definitions of diagnostic yield, which limits comparability across studies [6].

When interpreted in relation to lesion size, our findings are concordant with the existing literature. Sensitivity increased progressively and significantly across predefined size categories: from 23.3% in lesions ≤10 mm to 46.0% in lesions 11–20 mm (*p* = 0.001), reaching 62.3% in lesions >20 mm, suggesting a clear correlation between lesion diameter and diagnostic performance. This observation emphasizes that reported differences in ENB outcomes across studies should be contextualized by lesion size distribution. While recent consensus recommendations advocate for the use of a strict definition of diagnostic yield in studies of advanced bronchoscopy [9], the central clinical question in patients with suspected lung cancer remains whether malignancy has been reliably excluded. Even specific benign pathological findings may not eliminate residual concern for cancer and often require continued surveillance. Sensitivity for malignancy may represent a more clinically decisive measure of procedural performance in this specific context. Diagnostic yield is an important complementary outcome in studies of advanced bronchoscopy, but the strict ATS/CHEST definition could not be applied in the present study because detailed benign diagnoses were unavailable in the dataset.

### 4.3. Bronchus Sign and Other Variables

The presence of a bronchus sign was associated with a markedly higher sensitivity for malignancy compared with lesions without one (64.3% vs. 32.7%, *p* < 0.001). In multivariable analysis, it more than doubled the odds of a successful malignant diagnosis (aOR 2.53). This finding is consistent with prior studies and reinforces the bronchus sign as the most important anatomical predictor of successful bronchoscopic sampling [7,13].

The prevalence of a bronchus sign was strongly size-dependent, increasing substantially with lesion diameter. In the smallest lesion category (≤10 mm), a mere 11.1% of lesions exhibited a bronchus sign compared to 63.3% in lesions >20 mm, inhibiting meaningful subgroup analyses within this size group. This limitation is, however, informative in itself, as it indicates that absence of a bronchus sign is the rule rather than the exception in very small peripheral nodules. The combined effect of small lesion size and absence of bronchial access may account for the low sensitivity observed in the smallest lesions.

Other anatomical factors also influenced outcomes. Lesions located in the lower lobes demonstrated significantly lower sensitivity (35.8%) compared with upper or middle lobe lesions (48.1%). In multivariable analysis, lower lobe location remained independently associated with reduced sensitivity (aOR 0.35, *p* < 0.001). This finding is consistent with previous reports suggesting that lower lobe lesions may be more challenging to access with ENB, potentially due to exaggerated respiratory motion and navigational limitations [13].

Taken together, these findings underscore the importance of careful patient and lesion selection when considering ENB, especially for very small nodules.

### 4.4. Safety Profile

The overall complication rate was low, with pneumothorax occurring in fewer than 1% of procedures. This finding is consistent with the favorable safety profile of ENB reported in previous studies and supports its role as a minimally invasive diagnostic alternative to transthoracic biopsy [6]. It should be noted, however, that post-procedural chest radiography was performed only in cases of clinical suspicion. Consequently, small, asymptomatic pneumothoraces may have gone undetected, which should be considered when comparing complication rates across studies employing routine post-procedural imaging.

### 4.5. Technological Context and Temporal Considerations

All procedures were performed during 2018 and 2019 using the SuperDimension™ electromagnetic navigation system. Since then, ENB technology has evolved, with newer platforms incorporating improved positional feedback and navigation accuracy. In the randomized VERITAS trial, contemporary ENB achieved 79% diagnostic accuracy in 10–30 mm nodules and was non-inferior to TTNB [5]. As lesions <10 mm were not included, direct comparison with our cohort is limited. Nevertheless, the fundamental anatomical relationships identified here, particularly lesion size and bronchial access, are likely to remain relevant with newer technologies. Therefore, these anatomical principles are expected to remain key determinants of diagnostic success, supporting the continued relevance of our findings for contemporary ENB practice.

However, we believe that robotic bronchoscopy should be considered separately from conventional ENB. Recent studies of shape-sensing robotic bronchoscopy have shown high diagnostic performance in bronchus-sign-negative lesions, while the influence of lesion size has varied between studies [14,15]. These findings suggest that anatomical predictors identified with conventional ENB may differ in importance with robotic platforms.

### 4.6. Clinical Implications for Very Small Pulmonary Nodules

The markedly reduced sensitivity for malignancy (23.3%) observed in lesions ≤10 mm has important clinical implications. In this size category, the number needed to test to identify one malignant lesion becomes increasingly high, particularly in populations with low or intermediate pretest probability of malignancy. Under such conditions, routine ENB sampling of very small nodules may be inefficient and of limited clinical value. In the current cohort, 17 malignant diagnoses were obtained from 162 ENB procedures in the ≤10 mm subgroup, corresponding to approximately 9.5 procedures per malignant diagnosis.

For very small lesions that are technically amenable to sublobar resection and carry a high suspicion of malignancy, primary surgical management may be considered a more appropriate strategy than bronchoscopic biopsy in many cases [16,17]. This is supported not only by the low sensitivity observed in the present study, but also by accumulating evidence demonstrating favorable outcomes with sublobar resection in similar settings. Conversely, when the estimated malignancy risk does not justify immediate surgery for this category of lesions, short-interval radiological surveillance to assess growth may be preferable to invasive diagnostic procedures that carry a high false-negative rate. Taken together, these findings suggest that ENB may have limited utility as a primary diagnostic modality in very small nodules, particularly when pretest probability is low (favoring surveillance) or high (favoring definitive treatment). Although these considerations should be interpreted in light of the observational design of the present study, they highlight the necessity of integrating ENB results with pretest probability, anatomical characteristics, and alternative management strategies in individualized clinical decision-making. Future studies should further refine decision-making algorithms for the evaluation of very small pulmonary nodules.

### 4.7. Strengths and Limitations

Strengths of this study include the robust cohort size, the substantial representation of very small lesions (29.9% ≤ 10 mm), and the use of sensitivity for malignancy as the primary outcome, allowing direct quantification of the risk of missed cancer following ENB. Furthermore, the extended follow-up period enabled robust classification of diagnostic outcomes.

Limitations include the retrospective design and the single-center setting, which limit generalizability and may introduce bias. Not all anatomical variables of potential relevance were systematically available, meaning that residual confounding cannot be excluded. Multivariable analyses were based on complete cases, which may have introduced some degree of selection bias. The low prevalence of bronchus sign in the smallest lesion category limited subgroup analyses. Bronchus sign status was assessed by a single reviewer, precluding assessment of interobserver agreement. FDG-PET avidity was classified from clinical reports without a prespecified SUV threshold, which may have introduced misclassification and limits interpretation of its association with diagnostic sensitivity. Although evenly distributed across size categories, exclusion of indeterminate cases (mainly due to incomplete follow-up) may have introduced bias and influenced absolute sensitivity estimates. The broad false-negative definition may have introduced misclassification and resulted in conservative estimates of diagnostic sensitivity. A further limitation is that strict ATS/CHEST diagnostic yield could not be calculated because specific benign ENB diagnoses were not distinguished from nonspecific or nondiagnostic findings. Finally, the use of an earlier-generation ENB system may have influenced absolute performance metrics, although relative associations between key variables are likely preserved.

## 5. Conclusions

The sensitivity for malignancy in ENB is strongly associated with lesion size and bronchial access and is reduced in lower lobe lesions, highlighting important anatomical limitations. In very small nodules (≤10 mm), overall sensitivity is low, underscoring the need for careful patient selection and consideration of alternative management strategies.

## Figures and Tables

**Table 1:** Characteristics of Patients, lesions, and procedures.

Patients		
Age, median (IQR)		72 (66–76)
Female sex, no. (%)		317 (58.6)
ECOG Performance Status, median (IQR)		0 (0–1)
SVF participation, no. (%)		403 (74.5)
**Lesions**		
Total no.		541
Diameter, median (IQR) mm		13 (10–17)
Diameter, by group, no. (%)		
	≤10 mm	162 (29.9)
	11–20 mm	300 (55.5)
	>20 mm	79 (14.6)
Bronchus sign present, no. (%)		
	Overall	160 (29.6)
	≤10 mm	18 (11.1)
	11–20 mm	92 (30.7)
	>20 mm	50 (63.3)
Density, no. (%)		
	Solid	474 (87.6)
	Subsolid	67 (12.4)
FDG-PET hypermetabolism, no. (%)		342 (73.4)
Lobe location, no. (%)		
	Upper/middle lobe	361 (67.2)
	Lower lobe	176 (32.8)
**Procedures**		
Use of ROSE, no. (%)		246 (45.5)
>1 target lesion, no. (%)		124 (22.9)

IQR, interquartile range; ECOG, Eastern Cooperative Oncology Group; SVF, Swedish standardized diagnostic pathway for lung cancer; ROSE, rapid on-site examination. FDG-PET, fluorodeoxyglucose positron emission tomography; FDG-PET hypermetabolism is reported among lesions with available PET imaging (*n* = 466). Lobe location was available for 537 lesions; data were missing for 4 cases.

**Table 2:** Factors associated with sensitivity for malignancy.

**(a). Sensitivity stratified by lesion and procedural characteristics**						
**Characteristic**		**Malignant lesions, no.**	**TP**	**FN**	**Sensitivity, % (95% CI)**	***p*-value**
Bronchus sign						
	Yes	115	74	41	64.3 (54.9–73.1)	
	No	208	68	140	32.7 (26.4–39.5)	<0.001
Lesion density						
	Solid	282	128	154	45.4 (39.5–51.4)	
	Subsolid	41	14	27	34.1 (20.1–50.6)	0.235
FDG-PET avid						
	Yes	248	112	136	45.2 (38.9–51.6)	
	No	36	10	26	27.8 (14.2–45.2)	0.074
ROSE used						
	Yes	161	79	82	49.1 (41.1–57.1)	
	No	161	63	98	39.1 (31.5–47.1)	0.092
>1 target lesion						
	Yes	81	43	38	53.1 (41.7–64.3)	
	No	238	99	139	41.6 (35.3–48.1)	0.095
Lobe location						
	Upper/middle	214	103	111	48.1 (41.3–55.0)	
	Lower	106	38	68	35.8 (26.9–45.8)	0.0496
**(b). Independent predictors of diagnostic sensitivity**						
**Predictor**					**Adjusted odds ratio (95% CI)**	***p*-value**
Lesion size (per mm)					1.08 (1.03–1.14)	0.003
Bronchus sign present					2.53 (1.42–4.53)	0.002
Lower lobe					0.35 (0.19–0.62)	<0.001
Subsolid lesion					0.48 (0.21–1.07)	0.076
FDG-PET avid					1.03 (0.47–2.35)	0.938
ROSE used					1.15 (0.67–1.97)	0.600
>1 target lesion					1.48 (0.80–2.73)	0.207

Sensitivity was calculated among malignant lesions as TP/(TP + FN). In Table 2a, differences between groups were assessed using chi-square. Table 2b shows results from multivariable logistic regression with lesion size (included as a covariate to account for confounding) modeled as a continuous variable and other covariates as categorical. TP, true positive; FN, false negative; CI, confidence interval.

## Data Availability

The data presented in this study are not publicly available due to ethical and privacy restrictions. De-identified data may be made available from the corresponding author upon reasonable request.

## Supplementary Materials

The following supporting information can be downloaded at: https://www.mdpi.com/article/10.3390/arm94050067/s1, Table S1: Sensitivity analysis including indeterminate cases; Table S2: Classification of true-negative and false-negative outcomes.
