# Supplementary material for: Sensitivity for Malignancy with Navigation Bronchoscopy in Small Lung Nodules
Source: Adv Respir Med. 2026 Sep 17;94(5):67. doi: 10.3390/arm94050067 (PMC13600125; doi:10.3390/arm94050067)
Supplement: Supplementary file 1 [file arm-94-00067-s001.zip › arm-4493149-supplementary.pdf]

Supplementary material

Supplementary Table S1. Sensitivity analysis including indeterminate cases

Lesion size	Malignant cases, n	Indeterminate cases, n	Primary sensitivity, % (95% CI)	Worst-case sensitivity, % (95% CI)
Overall	323	56	44.0 (38.5–49.6)	37.5 (32.6–42.6)
≤10 mm	73	19	23.3 (14.2–34.6)	18.5 (11.1–27.9)
>10 mm	250	37	50.0 (43.6–56.4)	43.6 (37.7–49.5)
11-20 mm	189	31	46.0 (38.8–53.4)	39.5 (33.0–46.3)
>20 mm	61	6	62.3 (49.0–74.4)	56.7 (44.0–68.8)

Worst-case analysis assumes that all indeterminate cases represented malignant lesions missed by ENB and were therefore classified as false negatives. In the primary analysis, indeterminate cases were excluded.

Supplementary Table S2. Classification of true-negative and false-negative outcomes

Classification	Basis for classification	n
**True negative**	≥2 years of radiological follow-up without progression	77
	Significant radiological regression during follow-up	72
	Surgical resection with benign pathology	13
	**Total**	**162**
**False negative**	Surgical resection with malignant pathology	79
	Radiotherapy without histological confirmation	37
	Subsequent malignancy during follow-up*	65
	**Total**	**181**

*Includes malignancy of the target lesion confirmed by an alternative diagnostic procedure, subsequent lung cancer considered clinically or radiologically to correspond to the target lesion, and extrathoracic malignancy where the target lesion was considered consistent with pulmonary metastatic disease.
